# Skeletal muscle mass is associated with erythropoietin response in hemodialysis patients

**DOI:** 10.1186/s12882-021-02346-6

**Published:** 2021-04-16

**Authors:** Tomoaki Takata, Yukari Mae, Kentaro Yamada, Sosuke Taniguchi, Shintaro Hamada, Marie Yamamoto, Takuji Iyama, Hajime Isomoto

**Affiliations:** 1 Division of Gastroenterology and Nephrology, Faculty of Medicine, , Tottori University, 36-1 Nishimachi, Tottori 683-8504 Yonago, Japan; 2 Taniguchi Hospital, Kurayoshi, Japan

**Keywords:** ESA, Hyporesponsiveness, anemia, Muscle wasting, Sarcopenia, Resistance

## Abstract

**Background:**

Hyporesponsiveness to erythropoietin stimulating agent (ESA) is associated with poor outcomes in patients with chronic kidney disease. Although ESA hyporesponsiveness and sarcopenia have a common pathophysiological background, clinical evidence linking them is scarce. The purpose of the study was to investigate the relationship between ESA responsiveness and skeletal muscle mass in hemodialysis patients.

**Methods:**

This cross-sectional study analyzed 70 patients on maintenance hemodialysis who were treated with ESA. ESA responsiveness was evaluated by erythropoietin resistance index (ERI), calculated as a weekly dose of ESA divided by body weight and hemoglobin (IU/kg/week/dL), and a weekly dose of ESA/hemoglobin (IU/week/dL). A dose of ESA is equivalated to epoetin β. Correlations between ESA responsiveness and clinical parameters including skeletal muscle mass were analyzed.

**Results:**

Among the 70 patients, ERI was positively correlated to age (*p* < 0.002) and negatively correlated to height (*p* < 0.001), body weight (*p* < 0.001), BMI (*p* < 0.001), skeletal muscle mass (*p* < 0.001), transferrin saturation (TSAT) (*p* = 0.049), and zinc (*p* = 0.006). In the multiple linear regression analysis, TSAT, zinc, and skeletal muscle mass were associated with ERI and weekly ESA dose/hemoglobin.

**Conclusions:**

Skeletal muscle mass was the independent predictor for ESA responsiveness as well as TSAT and zinc. Sarcopenia is another target for the management of anemia in patients with hemodialysis.

## Background

Anemia is one of the major complications in patients with chronic kidney disease (CKD) or patients receiving hemodialysis (HD).It is related to poor outcomes [1, 2]. Erythropoietin stimulating agents (ESAs) have been used in patients with HD for many years [3, 4]. ESAs helps in achieving recommended hemoglobin levels. They are the most established agents for renal anemia [5, 6]. On the other hand, approximately 15 % of the patients are hyporesponsive to ESA [7, 8]. ESA hyporesponsiveness is associated with mortality and cardiovascular events in patients with CKD [9, 10]. Several conditions such as iron deficiency and zinc deficiency, which are easily treated, can cause ESA hyporesponsiveness. Therefore, it is necessary to look into the relevant cause when the patients show no or little response to ESA.

Among many factors that are associated with ESA hyporesponsiveness, malnutrition is one of the causes [11]. Deficiency of nutrients required for hematopoiesis, such as iron, zinc, vitamin B12, and copper, leads to ESA hyporesponsiveness. Infection and inflammation are the other causes for the hyporesponsiveness of ESA due to disturbance in iron utilization. Since each of these conditions requires different treatments, it is important to appropriately identify the relevant cause when the patients show no or little response to ESA.

Sarcopenia is characterized by loss of skeletal muscle mass that progresses with aging. It is being recognized as a great health issue in the elderly population [12, 13]. Growing evidence has revealed that sarcopenia is related to cardiovascular disease [14], cognitive function [15], physical performance [16], and mortality [17]. The pathogenesis of sarcopenia involves various conditions such as malnutrition and inflammation. These conditions are associated with erythropoietin resistance. In addition, recent studies demonstrated the associations between muscle mass and erythropoiesis [18, 19]. Although there is potentially an association between sarcopenia and ESA hyporesponsiveness, clinical evidence linking them is lacking. We hypothesized that muscle wasting is associated with ESA response, by reflecting nutritional and inflammation status. In the present study, we aimed to investigate the relationship between muscle mass and ESA hyporesponsiveness in hemodialysis patients receiving ESA.

## Methods

### Study population

This cross-sectional study included patients who had been on maintenance hemodialysis for at least 3 months at our hospital between April to June 2018. Patients with a history of amputation of extremities, with hemorrhagic lesions, and who did not reach the dry weight during the investigation were excluded from the study. Patients who were not treated with ESA were also excluded. All the patients were receiving hemodialysis/hemodiafiltration three times/week. Patient’s characteristics including the cause of end-stage renal disease, duration of hemodialysis, height, and body weight were collected from their medical records. Blood samples were collected at the beginning and the end of the dialysis session following a 2-day interval. Laboratory results at the end of each day for more than one month of stable ESA dose were used for the analysis. Erythropoietin resistance index (ERI) was calculated as a weekly dose of epoetin β divided by body weight and hemoglobin level (IU/kg/week/g/dL). Since the patients were treated with different ESAs, a dose conversion ratio of 1:200 for darbepoetin α and 1:225 for CERA were used for epoetin β [20, 21]. Patients with apparent iron deficiency, transferrin saturation (TSAT) < 20 % or ferritin < 100 ng/dL were treated with an injection of iron. The normalized protein catabolic rate (nPCR) and the dialysate dosage, the clearance of urea (K; mL/min) multiplied by the time on dialysis (t; min) divided by the volume of distribution (V; mL), were calculated as previously described [22]. This study was approved by the ethical committee of the Tottori University Hospital (approval number: 19A222) and conducted in accordance with the Declaration of Helsinki.

### Measurement of skeletal muscle

The skeletal muscle mass of each patients was measured by bioimpedance analysis (BIA) using InBody (InBody Japan, Tokyo, Japan). The measurement was performed after a session of hemodialysis to eliminate the influence of excess body fluid. The dry weight was determined according to their physical findings, chest radiograph, and serum brain natriuretic peptide or human atrial natriuretic peptide level. Skeletal muscle index (SMI) was calculated as skeletal muscle mass divided by height (kg/m^2^).

### Statistical analysis

The distribution of the continuous variables was evaluated using the Kolmogorov–Smirnov test. The variables were expressed as mean ± SD or median (range). Correlations between skeletal muscle mass or SMI and the patient’s characteristics were analyzed by Pearson’s correlation coefficient for normally distributed variables and Spearman’s correlation coefficient for non-normally distributed variables. Multiple linear regression analysis, in which sex, age, and laboratory findings were selected with stepwise forward selection method, was performed to investigate the influencing factor for skeletal muscle or SMI. A two-tailed p-value of less than 0.05 was considered statistically significant. Statistical analyses were performed using StatFlex (ver7.0 for Windows, Artec, Osaka, Japan) or GraphPad Prism (ver7.0 for Windows, GraphPad Software, San Diego, CA, USA).

## Results

### Patient characteristics

**Fig. 1:**
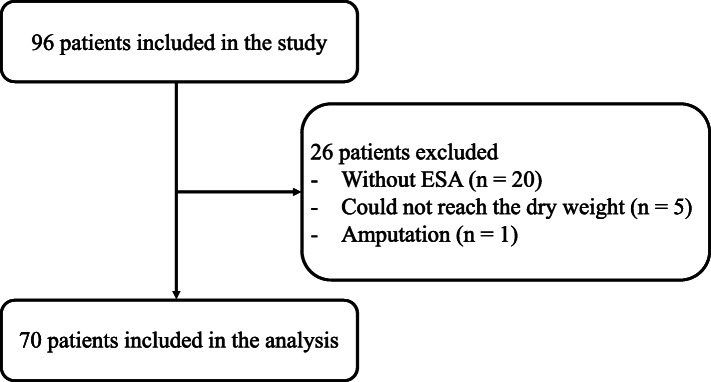
Study design. Seventy patients were included in the analysis and 26 patients were excluded

A total number of 96 patient records were reviewed in this study. Twenty-six patients were excluded (20 without ESA, 5 did not reach the dry weight, and 1 with amputation). Of the patients from the cohort, 70 (45 male and 25 female) were included in the analysis (Fig. 1). The characteristics of the study population are summarized in Table 1. The mean age of the participants was 67.2 ± 13.0 years, the mean ERI was 7.2 ± 5.4, and the mean skeletal muscle mass was 21.8 ± 5.4 kg.

**Table 1:** Patient's characteristics

	*N* = 70
Age, years	67.2 ± 13.0
Sex (male/female)	45 / 25
Duration of hemodialysis, months	216 (5-1219)
Height, m	1.60 ± 0.10
Body weight, kg	57.9 ± 13
BMI, kg/m^2^	22.3 ± 3.5
ERI, IU/kg/week/g/dL	7.2 ± 5.4
Skeletal muscle mass, kg	21.8 ± 5.4
Hemoglobin, g/dL	10.9 ± 0.9
Albumin, g/dL	5.6 ± 0.4
CRP, mg/dL	0.19 (0.05–3.25)
Calcium, mg/dL	8.6 ± 0.6
Phosphate, mg/dL	5.4 ± 1.3
Intact PTH, pg/mL	94 (5-887)
Magnesium, mg/dL	2.6 ± 0.3
TSAT, %	25.3 ± 12.1
Ferritin, ng/mL	79 (11–662)
Zinc, mg/dL	54.7 ± 9.0
Copper, mg/dL	98,3 ± 17.7
nPCR, g/kg/ideal body weight/day	0.82 ± 0.17
Kt/V urea	1.76 (0.98–2.99)

*BMI* body mass index; *ERI* erythropoietin resistance index; *CRP* C-reactive protein; *PTH* parathyroid hormone; *TSAT* transferrin saturation; *nPCR* normalized protein catabolic rate

### Correlations between ERI and clinical parameters

We first investigated the correlations between ERI and clinical parameters. ERI positively correlated to age (*p* < 0.002), and was negatively correlated to height (*p* < 0.001), body weight (*p* < 0.001), BMI (*p* < 0.001), skeletal muscle mass (*p* < 0.001), TSAT (*p* = 0.049), and zinc (*p* = 0.006). There were significant correlations between ERI and body size. Therefore, we further investigated the correlations between weekly ESA dose/hemoglobin and clinical parameters. As a result, we observed positive correlations with age (*p* = 0.020) and negative correlations with height (*p* = 0.017), body weight (*p* = 0.029), skeletal muscle mass (*p* = 0.011), TSAT (*p* = 0.009), and zinc (*p* = 0.013). These correlations are summarized in Table 2.

**Table 2:** Correlations between ESA response and clinical parameters

	ESA dose / Hb		ERI	
	r	*p* value	r	*p* value
Age	0.282	0.020	0.373	0.002
Duration of hemodialysis	0.057	0.65	0.116	0.35
Height	-0.288	0.017	-0.531	< 0.001
Body weight	-0.265	0.029	-0.565	< 0.001
BMI	-0.192	0.12	-0.455	< 0.001
Muscle mass	-0.307	0.011	-0.541	< 0.001
Albumin	-0.119	0.33	-0.173	0.16
CRP	-0.003	0.98	-0.027	0.83
Calcium	0.003	0.98	0.056	0.65
Phosphate	-0.096	0.43	-0.122	0.32
Intact PTH	-0.127	0.30	-0.147	0.23
Magnesium	0.077	0.58	0.018	0.90
TSAT	-0.317	0.009	-0.239	0.049
Ferritin	-0.135	0.27	-0.063	0.61
Zinc	-0.303	0.013	-0.331	0.006
Copper	0.040	0.75	0.016	0.89
nPCR	0.019	0.88	0.175	0.16
Kt/V	0.016	0.90	0.159	0.20

*SMI* skeletal muscle index; *BMI* body mass index; *PTH* parathyroid hormone; *CRP* C-reactive protein, *TSAT* transferrin saturation; *nPCR* normalized protein catabolic rate

### Correlations between skeletal muscle mass and clinical parameters

Correlations between skeletal muscle mass and clinical parameters were also investigated. Skeletal muscle mass was positively correlated with height (*p* < 0.001), body weight (*p* < 0.001), BMI (*p* < 0.001), albumin (*p* = 0.001), and zinc (*p* = 0.029), and negatively correlated with age (*p* < 0.001), nPCR (*p* = 0.028), and Kt/V (*p* < 0.001) (Table 3).

**Table 3:** Correlations between skeletal muscle mass and clinical parameters

	r	*p*-value
Age, years	-0.548	< 0.001
Duration of hemodialysis, months	-0.116	0.35
Height, m	0.857	< 0.001
Body weight, kg	0.799	< 0.001
BMI, kg/m^2^	0.514	< 0.001
Hemoglobin, g/dL	0.140	0.25
Albumin, g/dL	0.376	0.001
CRP, mg/dL	-0.116	0.35
Calcium, mg/dL	-0.211	0.079
Phosphate, mg/dL	0.106	0.38
Intact PTH, pg/mL	0.184	0.13
Magnesium, mg/dL	-0.103	0.45
TSAT, %	0.092	0.45
Ferritin, ng/mL	-0.113	0.35
Zinc, mg/dL	0.265	0.029
Copper, mg/dL	-0.151	0.22
nPCR, g/kg/ideal body weight/day	-0.273	0.028
Kt/V urea	-0.424	< 0.001

*BMI* body mass index; *ERI* erythropoietin resistance index; *CRP* C-reactive protein; *PTH* parathyroid hormone; *TSAT* transferrin saturation; *nPCR* normalized protein catabolic rate

### Determinants of skeletal muscle mass

Multiple linear regression analysis was performed to investigate the influencing factor for ERI. Age, sex, skeletal muscle mass, albumin, TSAT, intact PTH, Zinc, CRP, and Kt/V were selected as explanatory variables with the stepwise forward selection method. TSAT, zinc, and skeletal muscle mass were determined to be independent predictors for ERI (Table 4). Multiple linear regression analysis was also performed for weekly ESA dose/hemoglobin. TSAT, zinc, and skeletal muscle mass showed an independent association with weekly ESA dose/hemoglobin (Table 4).

**Table 4:** Multiple linear regression analysis

	Dependent variable			
	ESA dose/Hb		ERI	
	Stdβ	*p* value	Stdβ	*p* value
TSAT	-0.3422	0.003	-0.288	0.004
Zinc	-0.2665	0.022	-0.301	0.011
Muscle mass	-0.231	0.045	-0.533	< 0.001
CRP	-0.1807	0.11	-0.172	0.087

*SMI* skeletal muscle index; *BMI* body mass index. Stepforward selection method

## Discussion

In the present study, we observed that ESA responsiveness was associated with skeletal muscle mass. TSAT, zinc, and skeletal muscle mass are the independent predictors for ESA responsiveness.

ESA hyporesponsiveness is caused by various conditions. Iron deficiency is one of the major conditions leading to ESA hyporesponsiveness. It is recommended to measure TSAT and ferritin to assess the status of iron deficiency or iron overload. Serum ferritin is affected by inflammation, and TSAT is the most commonly used marker for the availability of iron [23]. Thus, we included TSAT in the multivariate analysis in the study. We found that TSAT is an independent predictor for ERI in line with the widely accepted recognition that iron deficiency causes ESA hyporesponsiveness. Inflammation can lead to the hyporesponsiveness of ESA [24]. Pro-inflammatory cytokines such as interleukin-6 increase the expression of hepcidin, which is the regulator of iron homeostasis [25, 26]. HD patients with inflammation showed increased hepcidin levels together with decreased intestinal absorption of iron [27]. A recent investigation in patients on hemodialysis showed that Malnutrition Inflammation Score, composite assessment of inflammation and nutritional status including serum albumin and transferrin, is associated with the response to ESA [28]. Although we did not find associations between CRP levels and ERI in the multivariate analysis, inflammatory conditions in our cohort might be, in some part, reflected in the iron status.

In the present study, we observed that skeletal muscle mass was associated with ERI. Previous observations showed associations between ERI and body composition including adipose tissue and muscle mass [29]. However, body size needs to be considered as an influencing factor for ERI. Since both skeletal muscle mass and ERI, calculated as a weekly dose of epoetin β divided by body weight and hemoglobin level, are closely related to body weight, we further analyzed the association between skeletal muscle mass and weekly ESA dose/hemoglobin to eliminate the influence of body size. We found that there was still an association between skeletal muscle mass and ESA responsiveness. Previous investigations in murine myoblast cells revealed that the erythropoietin receptor was expressed in myoblasts, and that erythropoietin promoted the proliferation of myoblasts [18]. Erythropoietin receptors are expressed in human skeletal muscle [19, 30]. In addition, muscle fibers can release erythropoietin after exercise, and erythropoietin-induced JAK2 phosphorylation, which is necessary to induce downstream signaling pathways of erythropoietin, increased after acute exercise [19]. On the other hand, long-term recombinant erythropoietin had no significant effecton muscle fiber hypertrophy [30]. These in vitro and human studies indicate that muscle mass is associated with ESA responsiveness and that muscle wasting is potentially a new target for managing anemia in patients with CKD.

We observed that zinc was also an independent predictor for ERI. Zinc deficiency is another cause of ESA hyporesponsiveness. The prevalence of zinc deficiency is extremely high in HD patients, and zinc supplementation reduces the dosage of erythropoietin [31]. Since most of the zinc distributes to skeletal muscle and bone [32], this might influence the correlation between skeletal muscle and ERI. However, we still found that skeletal muscle mass was associated with ERI independently of zinc.

There are some limitations to this study. ESA hyporesponsiveness is caused by various conditions that were not included in our study. Carnitine, vitamin, or folic acid are involved in ESA response. This is a retrospective study; thus, further investigation is required to determine whether exercise or intervention on skeletal muscle improves ESA responsiveness.

## Conclusions

In conclusion, we found that skeletal muscle mass is an independent predictor for ESA responsiveness as well as TSAT and zinc. Sarcopenia is another target for the management of anemia in patients with HD.

## Data Availability

The datasets used and/or analyzed during the current study are available from the corresponding author on reasonable request.

## Abbreviations

CKD: Chronic Kidney Disease
HD: Hemodialysis
ESA: Erythropoietin Stimulating Agent
ERI: Erythropoietin Resistance Index
BIA: Bioimpedance Analysis
SMI: Skeletal Muscle Index
TSAT: Transferrin Saturation
CRP: C-reactive Protein
